# Combined Effect of Subsurface Water Retention Technology and Arbuscular Mycorrhizal Fungi on Growth, Physiology and Biochemistry of Argan Seedlings under Field Conditions

**DOI:** 10.3390/plants13152098

**Published:** 2024-07-29

**Authors:** Boujemaa Fassih, Mohamed Ait-El-Mokhtar, Aicha Nait Douch, Abderrahim Boutasknit, Raja Ben-Laouane, Badia Aganchich, Said Wahbi

**Affiliations:** 1 Laboratory of Agro-Food, Biotechnologies and Valorization of Plant Bioresources (AGROBIOVAL), Department of Biology, Faculty of Science Semlalia, Cadi Ayyad University, Marrakesh 40000, Morocco; bo.fassih@gmail.com (B.F.); naitdouchaicha@yahoo.fr (A.N.D.); ba.aganchich@uca.ac.ma (B.A.); wahbi@ucam.ac.ma (S.W.); 2 Laboratory of Biochemistry, Environment & Agri-Food URAC 36, Department of Biology, Faculty of Science and Techniques—Mohammedia, Hassan II University of Casablanca, Mohammedia 28800, Morocco; 3 Department of Biology, Multidisciplinary Faculty of Nador, Mohammed Ist University, Nador 62700, Morocco; abderrahim.boutasknit@gmail.com; 4 Laboratory of Environment and Health, Department of Biology, Faculty of Science and Techniques, Errachidia 52000, Morocco; benlaouaneraja@gmail.com; 5 Centre d’Agrobiotechnologie et Bioingénierie, Unité de Recherche Labellisée CNRST (Centre AgroBiotech-URL-CNRST-05), Cadi Ayyad University, Marrakesh 40000, Morocco

**Keywords:** climate change, *Argania spinosa*, reforestation, sustainability, physiological leaf traits, biochemical response, soil fertility

## Abstract

The argan (*Argania spinosa* L. Skeels) ecosystem is severely degrading in arid and semi-arid lands due to climate change, particularly in terms of density loss and reforestation failure. Thus, it is important to adopt innovative effective sustainable practices to optimize the densification and reforestation success of the argan tree. The purpose of the present research was to investigate the combined effect of subsurface water retention technology (SWRT) and the use of native arbuscular mycorrhizal fungi (AMF) on edaphic, growth, physiological and biochemical parameters of field-grown argan seedlings in the Essaouira region, Morocco. In this experiment, one-year-old argan seedlings were transplanted in the absence and presence of biodegradable plastic and AMF. Our findings revealed that the application of SWRT enhanced soil profile moisture up to 640% at 40 cm depth compared to the control. The combination of this technology with AMF also improved soil fertility. Furthermore, the application of SWRT, with or without AMF, significantly enhanced argan seedling height (208 and 168%, respectively), stomatal conductance (54 and 33%, respectively), and chlorophyll fluorescence (21 and 20%, respectively). Similarly, the combined application of SWRT and AMF significantly improved protein and sugar content (36 and 57%, respectively), as well as antioxidant enzyme activities (peroxidase and polyphenol oxidase) and chlorophyll pigments content compared to the control. However, this treatment reduced malondialdehyde and H_2_O_2_ content in the argan leaves. As a summary, SWRT technology combined with AMF may be used as a valuable strategy to promote the success of argan reforestation and to limit soil erosion and desertification in arid and semi-arid climates.

## 1. Introduction

*Argania spinosa* L. Skeels is a tree species native to Morocco and holds significant importance in the national flora. It is the second most prevalent species by coverage, spanning an estimated 950,000 hectares [1,2,3,4]. This tree plays a crucial role in terms of its botanical, ecological, and socio-economical values especially for the local community [2,5]. The primary interest lies in its fruit, which yields highly valuable oil utilized not only for culinary purposes but also in cosmetics and medicine [2]. In 1998, it was designated as a Biosphere Reserve by UNESCO and May 10th has been designated as the international day of the argan tree by the United Nations [5].

Nowadays, the sustainability of the argan forest ecosystem is threatened by climate change due to its geographic location in the extremely arid southwest of Morocco [5]. This species is already suffering from ongoing land degradation resulting from continuous illegal firewood harvesting, excessive grazing pressure, and the argan tree’s slow growth [6]. Furthermore, the argan forest area was reduced during the last century, leading to a significant decline in terms of its density. The average density dropped from 100 to less than 30 trees per hectare [1]. In addition, it is difficult to successfully establish profitable argan plantations where many reforestation initiatives repeatedly fail, depriving nearby communities of essential grazing areas for extended periods [3]. These reforestation efforts are often hampered by the difficulty of getting plants to take root in the field, resulting in mediocre success rates for most plantations [7].

Reforestation failure is mainly due to the harsh environment, especially water availability as a limiting factor. Hence, there is a pressing need to find alternatives to promote reforestation success, such as water harvesting technologies. Subsurface Water Retention Technology (SWRT) is an innovative technology designed to create sustainable agricultural ecosystems from highly permeable soils with the implementation of an impermeable biodegradable membrane beneath the root zone [8]. It has demonstrated remarkable effectiveness in improving agricultural practices, particularly in light-textured soils. By installing specialized membranes in the root zone, SWRT efficiently retains water and nutrients, thus reducing water loss through deep percolation. Preserving these essential resources is crucial for ensuring the long-term viability of agricultural operations [9]. SWRT has been shown in numerous studies to improve plant performance and nutrient absorption, improve stomatal opening control for more effective plant water use, increase water supply by holding water near the root zone, and decrease oxidative damage under stressful conditions, particularly during drought [9,10,11,12].

The implementation of optimal biostimulant management techniques can also serve as means to alleviate climate change’s negative effects [13]. Arbuscular mycorrhizal fungi (AMF) are beneficial microorganisms that are important for the soil and can form symbiotic associations with nearly 78% of vascular plants, making it the most common symbiosis on the planet. This is one way to offset the negative effects of water deficiency [14]. Indeed, mycorrhizal association improves plant performance by promoting growth [15], water status, and nutrient accumulation during periods of drought [16]. According to several studies, AMF protect plants from water stress through a variety of mechanisms, such as morphological, physiological, and biochemical strategies [17,18].

To the best of our knowledge, this is the first study to discuss the impacts of SWRT and its combination with AMF on *Argania spinosa*. Therefore, this study’s aim is to find out more effective and sustainable practices to ensure the success of argan reforestation. In this context, we investigate the effects of two potentially beneficial agricultural technologies, SWRT and AMF, and apply both separately and in combination with a focus on their impact on soil moisture, as well as on the growth, physiology and biochemistry of field-transplanted argan seedlings in the Essaouira region.

## 2. Results

### 2.1. Soil Physicochemical Properties

The results showed that pH expedited a variation among treatments depending on the presence or absence of SWRT and/or mycorrhiza. Specifically, the soils from the four treatments exhibited pH values ranging between 7.89 and 8.10, with the highest values recorded in M and M + SWRT treated soil and the lowest one in Ct treatment (Table 1). However, the application of mycorrhiza alone or in combination with SWRT significantly increased electrical conductivity (EC) compared to the control, with increases of 31 and 43%, respectively. In addition, available phosphorus (AP) levels significantly increased in all treatments compared to the control, with the highest increase of 50% recorded in M + SWRT treatment. Furthermore, the treatments significantly increased total nitrogen (NTK) levels, with the greatest increase of 80% under M + SWRT followed by SWRT (69%) and then M (54%). Total organic carbon (TOC) has substantially increased in M and M + SWRT treatments compared to Ct with 26 and 51% increases, respectively, while SWRT showed a modest increase of 8%. Additionally, M and M + SWRT treatments enhanced organic matter (OM) levels by 27 and 51%, respectively, whereas SWRT exhibited a modest increase of 7%.

### 2.2. Soil Moisture Profile

**Figure 1:**
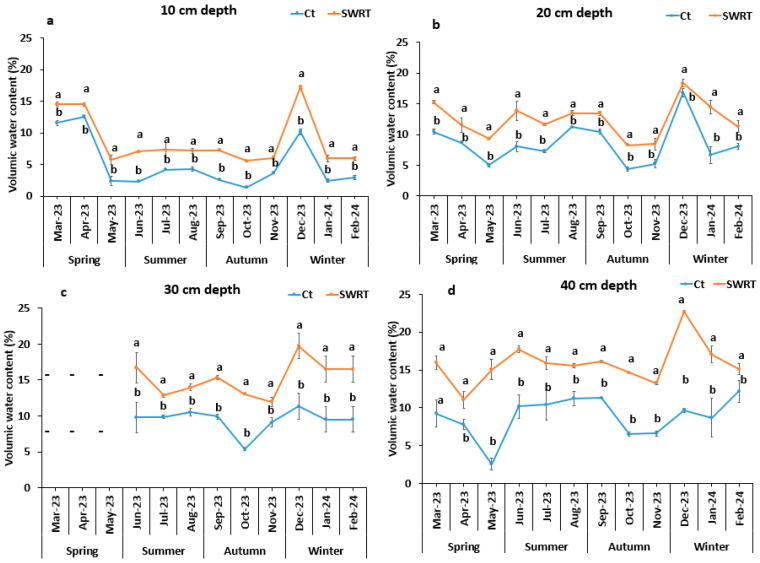
Monthly variation of soil moisture profile in the absence or the presence of subsurface water retention technology treatment at different soil depths: (**a**) 10 cm depth, (**b**) 20 cm depth, (**c**) 30 cm depth, (**d**) 40 cm depth. Ct: control, SWRT: subsurface water retention technology. Data denoted are means of five replicates (*n* = 5) ± standard error (SE). Different letters within the same month designate significant differences at *p* < 0.05 based on Tukey’s test.

The highest moisture profile values were recorded in the SWRT treatment compared with the control at all depths and during the study period (Figure 1). The most significant improvement of this parameter under SWRT treatment was recorded at 40 cm depth in December 2023 with a 640% increase in comparison to the Ct treatment, while the least significant increase (9%) was noticed at 20 cm depth during the same month.

### 2.3. Shoot Elongation

**Figure 2:**
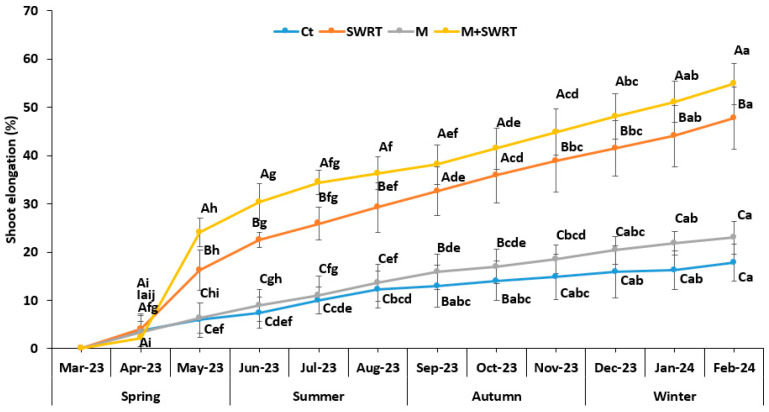
Monthly variation of shoot elongation in the absence and the presence of subsurface water retention technology and/or mycorrhiza. Ct: control; SWRT: subsurface water retention technology; M: mycorrhiza; M + SWRT: mycorrhiza + subsurface water retention technology. Data denoted are means of five replicates (*n* = 5) ± standard error (SE). Different capital letters within the same month indicate significant differences between treatments and different lower-case letters within the same treatment indicate significant differences between months at (*p* < 0.05) based on Tukey’s test.

Overall, shoot elongation values were significantly higher in SWRT, M, and M + SWRT treatments compared to the control. No significant differences were found between treatments just after seedlings were transplanted (March and April 2023). However, SWRT and M + SWRT treatments showed significant differences over the months up to February 2024, with an improvement of 168 and 208%, respectively, compared to the control (Figure 2).

### 2.4. Chlorophyll Fluorescence

**Figure 3:**
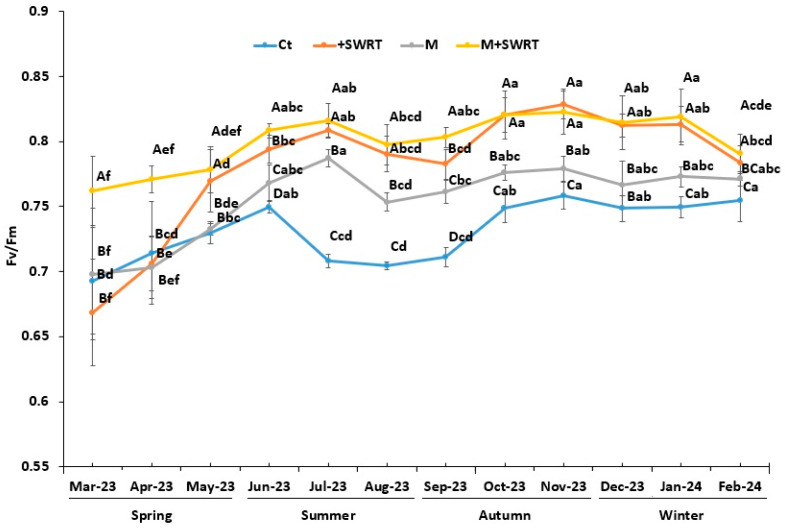
Monthly variation of argan seedlings chlorophyll fluorescence (Fv/Fm) in the absence and the presence of subsurface water retention technology and/or mycorrhiza. Ct: control, SWRT: subsurface water retention technology, M: mycorrhiza, M + SWRT: mycorrhiza + subsurface water retention technology. Data denoted are means of five replicates (*n* = 5) ± standard error (SE). Different capital letters within the same month indicate significant differences between treatments and different lower-case letters within the same treatment indicate significant differences between months at (*p* < 0.05) based on Tukey’s test.

Our results showed that the application of SWRT treatment alone and in combination with mycorrhiza had a significant effect on Fv/Fm compared to the Ct (Figure 3). Indeed, SWRT and M + SWRT treatments showed significant enhancement of this trait compared to the control throughout the year, with increase percentages ranging from 7 to 22%. The greatest increases for SWRT (19, 20, and 22%) were recorded in July, October, and November 2023, respectively, and the greatest ones for M + SWRT (19, 21, and 21%) were also recorded during the same period. However, the smallest percentages of increase were recorded for M treatment, ranging from 1 to 8%, offering a slight improvement over Ct.

### 2.5. Stomatal Conductance

**Figure 4:**
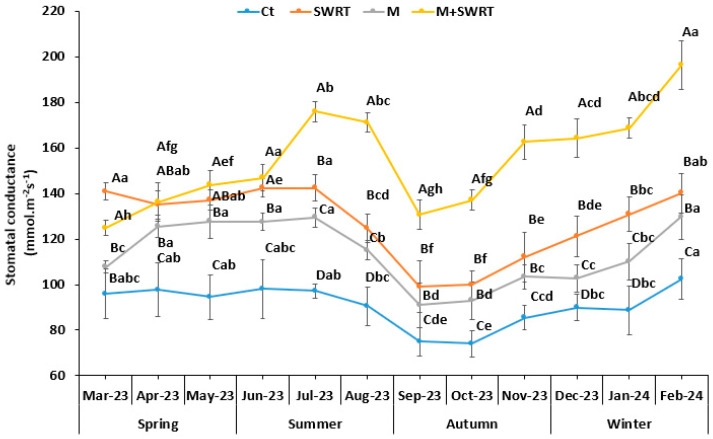
Monthly variation of argan seedlings’ stomatal conductance (gs) in the absence and the presence of subsurface water retention technology and/or mycorrhiza. Ct: control, SWRT: subsurface water retention technology, M: mycorrhiza, M + SWRT: mycorrhiza + subsurface water retention technology. Data denoted are means of five replicates (*n* = 5) ± standard error (SE). Different capital letters within the same month indicate significant differences between treatments and different lower-case letters within the same treatment indicate significant differences between months at (*p* < 0.05) based on Tukey’s test.

Stomatal conductance (gs) results showed a significant difference among the applied treatments and throughout the study period. Indeed, both SWRT and M + SWRT treatments showed the highest values of gs compared to the control (Figure 4), with the greatest increase percentages observed for M + SWRT treatment, ranging from 18 to 59% where the greatest increase was observed in February 2024. SWRT treatment came second and showed an almost steady increase compared to the Ct with the highest enhancement recorded in July 2023 (33%). Meanwhile, M treatment showed slight increases compared to the control, with percentages ranging from 11 to 21%, offering an improvement in comparison to the control, but less significant than M + SWRT and SWRT treatments.

### 2.6. Photosynthetic Pigments Content

**Figure 5:**
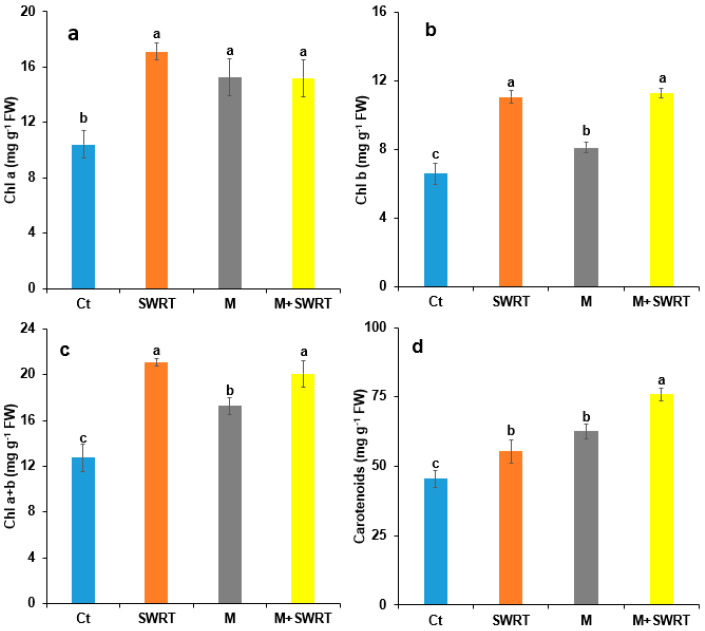
Effect of subsurface water retention technology and/or mycorrhiza treatments on argan seedlings’ photosynthetic pigments content (Chl *a* (**a**), Chl *b* (**b**), Chl *a* + *b* (**c**) and carotenoids (**d**)). Chl *a*: chlorophyll *a*; Chl *b*: chlorophyll *b*; Ch *a* + *b*: Total chlorophyll; Ct: control, SWRT: subsurface water retention technology, M: mycorrhiza, M + SWRT: mycorrhiza + subsurface water retention technology. Data denoted are means of three replicates (*n* = 3) ± standard error (SE). Different letters denote significant differences at *p* < 0.05 based on Tukey’s test.

Our findings showed that the application of SWRT and/or mycorrhiza showed significant increases compared to the control plants (Figure 5). Indeed, the highest values were recorded in plants treated with SWRT with 71% increase for Chl *a*, followed by the combined treatment (M + SWRT) with a 64% increase for Chl *b*. Regarding total chlorophyll, M + SWRT showed an increase of 67% followed by SWRT treatment with 68% of enhancement compared to the control plants. For carotenoids, the combined treatment also showed the highest level of increase (67%) followed by mycorrhiza (38%) and then SWRT (22%) treatments.

### 2.7. Malondialdehyde and Hydrogen Peroxide Content

**Figure 6:**
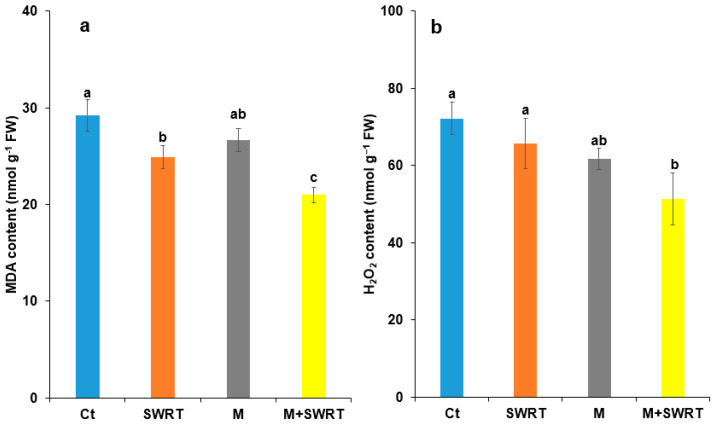
Effects of subsurface water retention technology and/or mycorrhiza treatments on argan seedlings’ (**a**) malondialdehyde and (**b**) H_2_O_2_ accumulations. Ct: control, SWRT: subsurface water retention technology, M: mycorrhiza, M + SWRT: mycorrhiza + subsurface water retention technology, MDA: malondialdehyde. Data denoted are means of three replicates (*n* = 3) ± standard error (SE). Different letters denote significant differences at *p* < 0.05 based on Tukey’s test.

Malondialdehyde (MDA) results reveal a significant difference between SWRT, M, M + SWRT treatments and Ct (Figure 6a). M + SWRT-treated argan plants showed the greatest decline in MDA (33%) followed by the SWRT treatment (20%) compared to the control, while the lowest percentage (10%) was recorded in the argan trees treated with mycorrhiza only. In terms of H_2_O_2_ content, our findings showed high significant differences between M + SWRT and Ct, although the SWRT treatment had no significant difference compared with the control (Figure 6b). On the other hand, M + SWRT showed a significant reduction of 14% compared to the control, followed by M with 20% reduction compared to the control.

### 2.8. Protein and Soluble Sugars Accumulation

**Figure 7:**
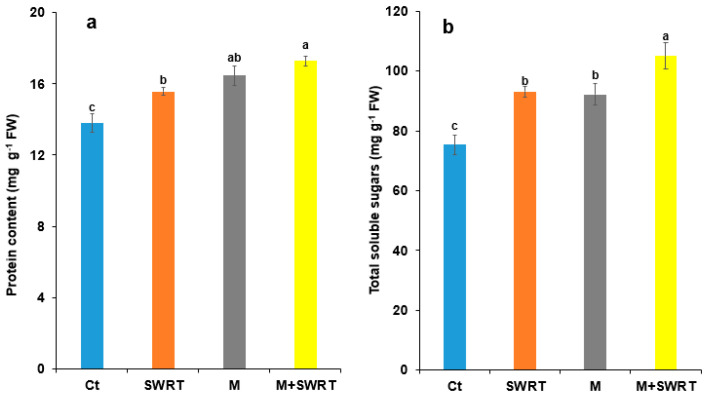
Effects of subsurface water retention technology and/or mycorrhiza treatments on argan seedlings’ (**a**) proteins and (**b**) total soluble sugars contents. Ct: control; SWRT: subsurface water retention technology; M: mycorrhiza; M + SWRT: mycorrhiza + subsurface water retention technology. Data denoted are means of three replicates (*n* = 3) ± standard error (SE). Different letters denote significant differences at *p* < 0.05 based on Tukey’s test.

Data presented in Figure 7 reveal that protein and sugars contents exhibited a significant variation between the applied treatments. M + SWRT treatment demonstrated a significant difference for protein and soluble sugars compared to the control. Moreover, the highest protein content was recorded in M + SWRT-treated argan seedlings with an enhancement of 36% over the control, whereas the lowest value of this trait was recorded in SWRT-treated argan seedlings with an increase of 18% over the control. On the other hand, soluble sugars content displayed the highest increase under M + SWRT treatment with 57% enhancement, while the lowest improvement was recorded for M treatment (22%) compared to the Ct.

### 2.9. Antioxidant Enzymes Activity

**Figure 8:**
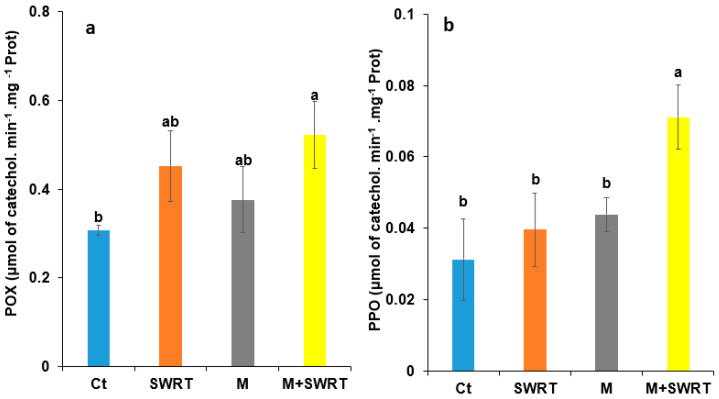
Effects of subsurface water retention technology and/or mycorrhiza treatments on argan seedlings’ (**a**) peroxidase and (**b**) polyphenol oxidase activities. Ct: control; SWRT: subsurface water retention technology; M: mycorrhiza; M + SWRT: mycorrhiza + subsurface water retention technology; PPO: polyphenol oxidase; POX: peroxidase. Data denoted are means of three replicates (*n* = 3) ± standard error (SE). Different letters denote significant differences at *p* < 0.05 based on Tukey’s test.

Our results showed significant variation of polyphenol oxidase (PPO) and peroxidase (POX) activities depending on the presence or absence of SWRT and/or mycorrhizae (Figure 8). The combined treatment (M + SWRT) displayed a significant increase over the Ct for both PPO and POX activities with increases of 129 and 70%, respectively, compared to the control. However, the separate application of SWRT and M treatments showed no significant increases compared to the control for both parameters.

## 3. Discussion

Drought and desertification significantly impact ecosystem sustainability, particularly in arid and semi-arid regions, which are among the most vulnerable [19]. In the Mediterranean areas, reforestation is limited, due to delayed rainfall, high evaporation, and soil degradation [19,20,21]. Furthermore, these regions’ soils are known for their low capacity to retain water, decreased microbiological biodiversity, and a lack of nutrients and organic matter [22]. To successfully reforest these degraded areas, it is crucial to identify cost-effective techniques that help plants mitigate the effects of drought and improve soil quality. As far as we know, this study is the first to explore the impact of SWRT and its combination with AMF on argan seedlings under field conditions. It explores the effects of SWRT, AMF, and their combination on soil quality and argan seedling growth, physiology, and biochemistry.

Our results demonstrate that using a native AMF consortium, either alone or in combination with SWRT, improves soil mineral nutrient levels and pH. The pH increase observed under SWRT treatment is likely due to enhanced soil texture and increased biological activity, as demonstrated by previous studies [22,23]. Conversely, M and M + SWRT treatments led to increased EC, suggesting a potential salt accumulation, possibly due to the reduced leaching under these conditions [24]. These findings are consistent with those of Belayneh et al. [23], who reported similar changes in soil properties following the implementation of soil conservation measures. Soil AP levels significantly increased under M and M + SWRT treatments. The obtained result can be explained by the presence of AMF in the soil. In fact, the application of AMF can induce a better availability of nutrients, including phosphorus, even in the presence of a high pH, for several reasons. By forming extensive mycelial networks in the soil, mycorrhizae considerably increase the absorption rate of phosphorus, thus enriching the rhizospheric area with AP, giving plants access to otherwise inaccessible forms of phosphorus [25]. In addition, some mycorrhizae secrete organic acids that can locally acidify the rhizosphere. This local acidification can increase the solubilization of phosphorus, making it more available to plants [26]. In addition, AMF can produce enzymes such as phosphatases, which release phosphorus from organic compounds in the soil [27]. Finally, AMF can also interact with calcium ions in the soil. By complexing this element, they reduce the formation of insoluble calcium phosphate complexes, thereby increasing the availability of phosphorus [28]. The findings of Lahbouki et al. [11] support this result, demonstrating that SWRT application significantly influences soil phosphorus availability. Soil total nitrogen levels also showed substantial increases under different treatments. These increases can be attributed to the accumulation of organic matter and enhanced microbial activity, which improve nitrogen mineralization. This is consistent with the results of previous studies [11,22] that found a similar increase in total nitrogen following the application of SWRT and/or AMF. Organic matter exhibited higher increase in the combined treatment. This result indicates that the dual application of SWRT and AMF induces a significant improvement in soil quality and its ability to retain nutrients and water and is in accordance with other studies [11,22]. Soil moisture findings indicate that the most significant improvement due to the application of SWRT was observed at 40 cm in depth. This treatment demonstrated a significant positive effect on soil water content at various depths throughout the year, enhancing the soil’s water-holding capacity. The same result is reported by Aoda et al. [10], who found that the application of SWRT improves water efficiency in the root zone at 15 and 30 cm depth through the use of biodegradable plastic since it promotes water retention and reduces its leaching.

According to the obtained results, it appears that the application of SWRT alone or in combination with mycorrhiza significantly enhanced shoot elongation of the argan seedlings. This finding is in accordance with the results of a recent study reporting that shoot height had been improved after the application of SWRT in tomato [11]. This enhancement could be explained by the increase of soil fertility (AP, NTK, and OM) and structure, as demonstrated by our study, and the decrease in water and mineral loss by percolation which promote an establishment environment for the promotion of plant growth processes.

On the other hand, Fv/Fm and gs showed seasonal patterns with highest values recorded in SWRT- and M + SWRT-treated argan seedlings, while those treated only with mycorrhizae exhibited the lowest values compared to the untreated seedlings. The same result was reported by Lahbouki et al. [11] who showed that the application of SWRT enhanced gs and Fv/Fm in tomato plants under field conditions. Many studies have reported that SWRT application increased the soil’s ability to hold water by providing a steady supply of accessible moisture and also traps more nutrients, which are crucial in boosting stomatal conductance and aperture [8,24,25]. Baslam et al. [29] suggested that the improvement in physiological traits, including plant water status, imply improved photosynthetic machinery performance, which results in increased CO_2_ uptake for photosynthesis. Furthermore, Naouraz et al. [30] reported that the higher values of photosynthetic efficiency of AMF-inoculated plants grown in soil amended with hydrogels demonstrated that the photosynthetic apparatus of these plants was more efficient in a drought stress situation.

In addition, AMF-inoculated plants enhanced the PSII efficiency and gs compared with the control plants, which corroborates previous studies [27,28] suggesting that AMF could enhance water status under water deficit conditions. According to Ye et al. [31], Fv/Fm of AMF-inoculated grapevine plants was enhanced by 40% in comparison with non-inoculated plants under water scarcity. It has been documented that AMF are beneficial, under water deficiency conditions, primarily to the growth, physiology, and absorption of nutrients in a variety of tree species, such as date palm [32], grape vine [31], and cocoa [33] plants. This mutual association of AMF with a plant increases root size and capacity, stomatal conductance and gas exchange, and also helps the plant to cope with adverse climatic conditions. AMF induce ABA responses controlling plant physiology and stomata movement. AMF-associated plants grown in arid areas respond to water scarcity through morphological adaptations that involve physiological and biochemical mechanisms. Furthermore, by releasing glomalin into the soil, AMF maintain plant-soil water relations and improve soil structure, which promotes stomatal conductance and photosynthesis efficiency [34].

This study’s findings also revealed that the application of SWRT and/or AMF on *Argania spinosa* seedlings significantly enhanced their chlorophyll and carotenoids contents compared to the control plants. The study by Lahbouki et al. [11] demonstrated that SWRT application increased photosynthetic pigments content in field-grown tomatoes. According to the same study, this could be attributed to the improvement of soil moisture after using SWRT which is in accordance with the soil moisture results. Furthermore, it has been reported that inoculation with mycorrhizae has a crucial compensatory effect on chlorophyll in arid conditions [35]. Zhu et al. [36] also reported that mycorrhizal *Zea mays* grown under water deficiency have far higher amounts of chlorophyll than the control. In the same vein, Zaouchi et al. [37] showed that mycorrhizal symbiosis improves plants’ photosynthetic efficiency and lessens the detrimental effects of water scarcity on the PSII reaction center, which leads to a relative rise in the concentration of chlorophyll pigments. The promoting effect of AMF on phosphorus and nitrogen assimilation by plants could also explain this increase in photosynthetic pigments content in AMF-treated argan seedlings [22].

Plants accumulate multiple types of compatible solutes, such as proteins and soluble sugars, to maintain their membrane integrity, control their plasma osmotic potential, and protect themselves from dehydration and oxidative damage under water deficit conditions [27,31,32]. Our findings showed that the application of SWRT and/or AMF improved protein and total soluble sugars content in treated argan seedlings compared to the control. These results are in accordance with previous studies [11,12] on tomato and cactus subjected to water deficiency. Indeed, Lahbouki et al. [11] reported that the application of SWRT showed a positive increase of protein content in tomato’s roots, shoots, and fruits. In another study, conducted by the same authors, soluble sugars and protein contents were enhanced in cactus pads [12]. In addition, Ye et al. [31] reported that the production of compatible organic solutes including proteins and sugars increased in *Vitis vinifera* plants inoculated with AMF. This can be explained by osmotic adaptation, which involves accumulating high levels of total soluble sugars and proteins. This accumulation helps maintain cell turgor and mitigate oxidative damage caused by water scarcity stress. Sharma et al. [38] suggested that organic osmolytes accumulation is associated with plant adaptation to water stress, as the photosynthates and plant hormones produced can adjust and reduce leaf osmotic potential, resulting in improved water uptake under drought stress.

It is well known that drought induces elevated levels of reactive oxygen species (ROS) at the cellular level, resulting in significant cellular damage and metabolic disruption [33,34]. As a result of this oxidative stress, plants significantly increase the accumulation of MDA and H_2_O_2_ [39]. MDA is synthesized within plant cell membranes through the breakdown of polyunsaturated fatty acids under dehydrating conditions, making it a recognized indicator of oxidative stress in plants [40]. Our findings showed that the application of SWRT alone or in combination with AMF significantly reduced MDA content. Our results are consistent with those of a previous study [11] where the authors reported that the application of SWRT on tomatoes prevented oxidative stress damage, shielding the cell membranes from damage through its capacity to increase soil water-holding capacity in the plant root zone and improve soil quality through increased AP, TOC, and OM. Ye et al. [31] also reported that AMF inoculation reduced MDA and H_2_O_2_ accumulation in *Vitis vinifera* L. under drought conditions. It was also found that the application of both SWRT and AMF significantly reduced H_2_O_2_ levels in the argan seedlings. Thus, the damage to the cell membrane caused by ROS, particularly H_2_O_2_, was lower in the treated argan seedlings than that in the untreated ones. It has been reported that AMF application decreased H_2_O_2_ and MDA concentrations in sweet scented geranium and trifoliate orange [36,37]. According to Boutasknit et al. [41], lower levels of H_2_O_2_ and MDA accumulation indicate a reduction in cell membrane oxidative damage under water stress.

To prevent cell damage from ROS accumulation and maintain homeostasis under drought conditions, plants evolve a powerful antioxidant enzyme system [11,33]. The ROS balance is maintained in cells by directly scavenging superoxide radicals (O_2_^−^) and H_2_O_2_ into less reactive species by several antioxidant enzymes acting in synchrony, such as PPO and POX [38,42,43]. Findings of the present study showed that argan seedlings exhibited significantly higher PPO and POX activities under M + SWRT treatment compared to the untreated seedlings. The same result was reported by Boutasknit et al. [44] who reported higher antioxidant enzyme activities (POX and PPO) in carob seedlings inoculated with AMF under water deficiency. A recent study [12] also found that the application of SWRT enhanced POX activity in *Opuntia ficus-indica* (L.) under limited irrigation conditions compared to the control. According to Dumanović et al. [45], enhanced activities of antioxidant enzymes facilitate the removal of ROS, thus enhancing plant resistance to water scarcity.

## 4. Materials and Methods

### 4.1. Experimental Site and Design, Plant Material, and Treatments

The field experiment was conducted in Id Bouzid douar (31°19′29.3″ N 9°32′32.8″ W, 360 m above sea level), Sidi Eljazouli commune, located 30 km southeast of Essaouira, Morocco. The region’s climate is semi-arid. The meteorological data of this site during the experiment duration are presented in Figure S1. The experimental plot extends over 2.7 hectares and is equipped with a natural organic farming system adopted without the use of herbicides or chemical fertilizers. The tillage was carried out under identical conditions for all the applied treatments, thus ensuring uniformity in experimental procedures. All weeds were removed manually when necessary. The physicochemical analyses of the soil prior transplanting were as follows: sand: 34.58%; silt: 48.51%; clay: 16.84%; AP: 38.40 ppm; TOC: 0.51%; TOM: 0.87%; pH: 7.39, and EC: 282 µS cm^−1^.

One-year-old argan seedlings of the Essaouira ecotype were transplanted in the field. Four treatments were included in the experimental design: the control treatment (Ct) without SWRT and AMF application, the SWRT treatment (SWRT), the AMF treatment (M) and the combination of AMF and SWRT (M + SWRT treatment). Treatment distribution was totally randomized and each treatment had 25 replicates, for a total of 100 plants.

SWRT technology was applied by burying a square meter of biodegradable plastic (a lifespan of 36 months and a thickness of 80 μm) at a depth of 60 cm, creating a U-shaped structure under each plant. In addition, a native AMF consortium was collected from the argan rhizosphere in the Essaouira region and then inoculated, after its multiplication using maize culture, into the rhizosphere soil of each argan seedling. Inoculation was carried out by adding 30 g of inoculum, comprising hyphae, vesicles, roots, and a substrate containing spores. Argan seedlings not inoculated with AMF received an equal amount of autoclaved mycorrhizal inoculum. Implementation of the field experiment began in February 2023 and the transplanted argan seedlings were irrigated by filling the microbasin of each seedling at a rate of 50 L per month.

### 4.2. Soil Measurements and Volumetric Water Content

One year after transplantation of the argan seedlings, soil samples were collected near the roots and were analyzed for their physicochemical characteristics. To measure soil pH and EC, 5 g of 2 mm sieved soil was weighed and added to 25 mL of distilled water. The diluted soil suspension (1:5 *v*/*v* ratio) was agitated for 30 min, then pH and EC measurements were made using a pH meter and a conductivity meter, respectively. Soil TOC content was determined using the Aubert method [46]. An amount of 1 g of sieved soil was homogenized in 10 mL of potassium bichromate K_2_Cr_2_O_7_ (1N) and 20 mL of concentrated sulfuric acid. After shaking for one minute and letting stand for 30 min, the reaction was stopped by adding 100 mL of distilled water. After 2 h of decanting, 25 mL of the solution was added to 5 mL of orthophosphoric acid and 3 drops of diphenylamine. After stirring, the bichromate excess was titrated with a solution of iron sulfate and ammonium until turning green. TOM content was determined by applying the following formula: TOM (%) = COT × 1.724. AP concentration measurement was based on the formation and reduction of a complex of orthophosphoric acid and molybdic acid as described by Olsen and Sommers [47]. Phosphorus-molybdate reduction is accompanied by a sky-blue coloration whose intensity is proportional to the amount of phosphorus present in the medium. The absorbance was measured using spectrophotometer at 820 nm. Total nitrogen (NTK) content was assessed using the Kjeldahl method [48] with an automated Kjeldahl distiller (KJA-9840 Model, Shandong, China). The volumetric water content in the soil was assessed monthly using the PR2 Profile Probe (Delta-T Devices, Cambridge, UK) at different depths (10, 20, 30, and 40 cm).

### 4.3. Physiological Measurements

The measurements of maximal photochemical efficiency of PSII (Fv/Fm) were made on fully expanded and grown leaves using a fluorometer (Opti-sciences OSI 30p, Hudson, NY, USA). Prior to measurement, the leaves were allowed to acclimate to darkness for 20 min using clips (five measures per treatment). The PSII efficiency was measured as Fv/Fm, where Fv is variable fluorescence (Fv = Fm − F0), Fm is maximum fluorescence, and F0 is initial fluorescence [49].

Stomatal conductance (gs) measurements were performed using a portable porometer (Leaf Porometer LP1989, Decagon Device, Inc., Washington, DC, USA) on the abaxial surface of fully grown and mature argan leaves. These measurements were performed in the morning (from 9:00 a.m. to 11:00 a.m.) on sunny days from the same row in the upper part of the plant. Five measurements were conducted for each treatment.

Photosynthetic pigments were extracted according to the Arnon method [50]. Briefly, 50 mg of crushed leaves was homogenized in 4 mL of 80% acetone. The homogenate was centrifugated for 10 min at 10,000× *g* and the absorbance of the supernatant was measured by a spectrophotometer (Spectrophotometer UV-3100PC) at the wavelengths of 663, 645, and 480 nm for chlorophyll *a* (Chl *a*), chlorophyll *b* (Chl *b*), and carotenoids (Car), respectively. The concentrations were calculated using the following equations:

$$Chlorophyll\;a\;\left(\frac{mg}{g}\right)=\left[\left(12.7\times A663\right)-\left(2.69\times A645\right)\right]\times \frac{V}{1000}\times FW$$

$$Chlorophyll\;b\;\left(\frac{mg}{g}\right)=[(22.9\times A645)-(4.68\times A663)]\times \frac{V}{1000}\times FW$$

$$Total\;Chlorophyll\;\left(\frac{mg}{g}\right)=[A480+(0.114\times A663)-(0.638\times A645)]\times \frac{V}{1000}\times FW$$

where V is final volume of the extract, and FW is fresh weight.

### 4.4. Biochemical Leaf Traits Measurement

#### 4.4.1. Quantification of Total Soluble Sugars

The total soluble sugars content was measured on fully expended argan leaves according to Dubois et al. [51]. An amount of 0.1 g of frozen leaves was mixed with 8 mL of ethanol (80%, *v*:*v*) then centrifugated for 10 min at 5000 rpm. A volume of 200 µL of obtained supernatant was mixed with 200 µL of phenol (5%) and 1 mL of sulfuric acid. After agitation and cooling for 15 min, the absorbance was read by spectrophotometer (Spectrophotometer UV-3100PC, VWR, Rosny-sous-Bois, France) at 485 nm.

#### 4.4.2. Quantification of Malondialdehyde and Hydrogen Peroxide

The concentration of MDA was measured according to the protocol of Savicka and Škute [52]. After extracting lipid peroxides from 0.25 g of frozen powder subsamples, 10 mL of 0.1% (*w*/*v*) trichloroacetic acid (TCA) was added. The extract was then centrifugated for 20 min at 18,000× *g*. A volume of 1 mL of the obtained supernatant was added to 2.5 mL thiobarbituric acid (TBA), leading to chromogen formation. After 30 min of incubation at 95 °C, the tubes were placed in an ice bath to halt the process. The absorbance (A) was read at 450 (A450), 532 (A532), and 600 (A600) nm. MDA content was determined using the formula below:

[MDA]=6.45×(A532−A600)−0.56×A450

The quantification of H_2_O_2_ was determined by the protocol of Velikova et al. [53]. An amount of 0.25 g of frozen crushed leaves was mixed with 5 mL of TCA 10% (*w*/*v*). After centrifugation at 15,000× *g* for 15 min, 0.5 mL of obtained supernatant was homogenized in 0.5 mL of potassium phosphate buffer (10 mM, pH 7) with the addition of 1 mL of potassium iodate (1 M). The absorbance was measured at 390 nm following a one-hour dark incubation period. A H_2_O_2_ standard curve was used to determine H_2_O_2_ concentrations.

#### 4.4.3. Quantification of Total Soluble Proteins and Antioxidant Enzyme Activities

In a cold mortar, 0.1 g of leaf samples were homogenized in 4 mL of 1 M phosphate buffer (pH 7) including 5% polyvinylpolypyrrolidone (PVPP) and 0.1 mM ethylenediaminetetraacetic acid (EDTA). After centrifuging the homogenate at 18,000× *g* for 15 min at 4 °C, the protein content and the antioxidant enzyme activities were measured using the supernatant. The amount of total soluble proteins was determined using Bradford’s technique [54]. PPO activity was measured following the method of Gauillard et al. [55] using catechol oxidation monitoring at 410 nm. The solution used included enzyme extract (0.1 mL), catechol (50 mM), and K_2_HPO_4_/KH_2_PO_4_ buffer (100 mM, pH 6). POX activity was evaluated according to the method of Tejera García et al. [56]. The reaction mixture consisted of K_2_HPO_4_/KH_2_PO_4_ buffer (100 mM), guaiacol (40 mM), H_2_O_2_ (10 mM), and the enzyme extract (0.1 mL). PPO and POX activities were expressed in unit mg protein^−1^.

### 4.5. Statistical Analysis

The data shown here represent the mean values ± standard error (S.E.). These values were obtained based on five replicates for soil moisture profile and argan growth and physiological traits and three replicates for biochemical analyses. Statistical analysis was performed using analysis of variance (ANOVA) using the SPSS software package version 25.0 (IBM, Armonk, NY, USA) for Windows. Tukey’s test was conducted at a significance level of *p* ≤ 0.05 to compare the mean values of the measured parameters under different treatments.

## 5. Conclusions

This study highlights the significant positive effects of SWRT and its combination with AMF on soil quality and on growth, physiological, and biochemical parameters of *Argania spinosa* seedlings. SWRT application increased soil moisture at different depths and improved its nutrient content when applied alone or in combination with AMF. In addition, both treatments boosted argan seedling height, photosynthesis efficiency, stomatal conductance, and photosynthetic pigments content. Our findings also revealed that the application of SWRT, either alone or in combination with AMF, prevented oxidative stress damage by reducing H_2_O_2_ and MDA accumulations and increasing the accumulation of proteins and sugars. Furthermore, these treatments enhanced the activities of antioxidant enzymes, facilitating the removal of ROS. Our findings strongly suggest that utilizing SWRT in conjunction with AMF can effectively promote the survival of the argan seedlings under drastic conditions in arid and semi-arid climates. This approach is highly recommended for successful reforestation efforts in the argan ecosystems. Further research will be performed during this study project, including the investigation of the different levels of the molecular aspect orchestrating the improvement of argan fitness and performance in semi-arid and arid environments in the presence of SWRT and AMF.

## Figures and Tables

**Table 1:** Soil physicochemical characteristics before and after one year of argan seedling transplantation.

	pH	EC (µS cm^−1^)	AP (ppm)	NTK (g Kg^−1^)	TOC (%)	OM (%)
Ct	7.89 ± 0.06 b	237.33 ± 6.02 c	31.46 ± 0.30 b	3.06 ± 0.32 c	0.39 ± 0.03 c	0.67 ± 0.05 c
SWRT	7.99 ± 0.06 ab	205.66 ± 5.03 d	40.60 ± 2.27 a	5.17 ± 0.03 a	0.42 ± 0.00 c	0.72 ± 0.00 c
M	8.10 ± 0.02 a	312.00 ± 4.58 b	43.40 ± 4.34 a	4.72 ± 0.06 b	0.49 ± 0.02 b	0.85 ± 0.03 b
M + SWRT	8.07 ± 0.01 a	340.66 ± 8.50 a	47.06 ± 2.11 a	5.51 ± 0.08 a	0.59 ± 0.03 a	1.01 ± 0.05 a

Ct: control; SWRT: subsurface water retention technology; M: mycorrhiza; M + SWRT: mycorrhiza + subsurface water retention technology; EC: electrical conductivity; AP: available phosphorus; NTK: nitrogen total Kjeldahl; TOC: total organic carbon; OM: organic matter. Data denoted are mean of three replicates (*n* = 3) ± standard error (SE). Different letters within the same column denote significant differences at *p* < 0.05 based on Tukey’s test.

## Data Availability

Data are contained within the article and Supplementary Materials.

## Supplementary Materials

The following supporting information can be downloaded at: https://www.mdpi.com/article/10.3390/plants13152098/s1, Figure S1: Variation of the monthly precipitations, temperatures (Tmean, T max and Tmin), and relative humidity during the experiment period.
