# Supplementary material for: Combined Effect of Subsurface Water Retention Technology and Arbuscular Mycorrhizal Fungi on Growth, Physiology and Biochemistry of Argan Seedlings under Field Conditions
Source: Plants (Basel). 2024 Jul 29;13(15):2098. doi: 10.3390/plants13152098 (PMC11313992; doi:10.3390/plants13152098)
Supplement: Supplementary file 1 [file plants-13-02098-s001.zip › plants-3095300-supplementary.pdf]

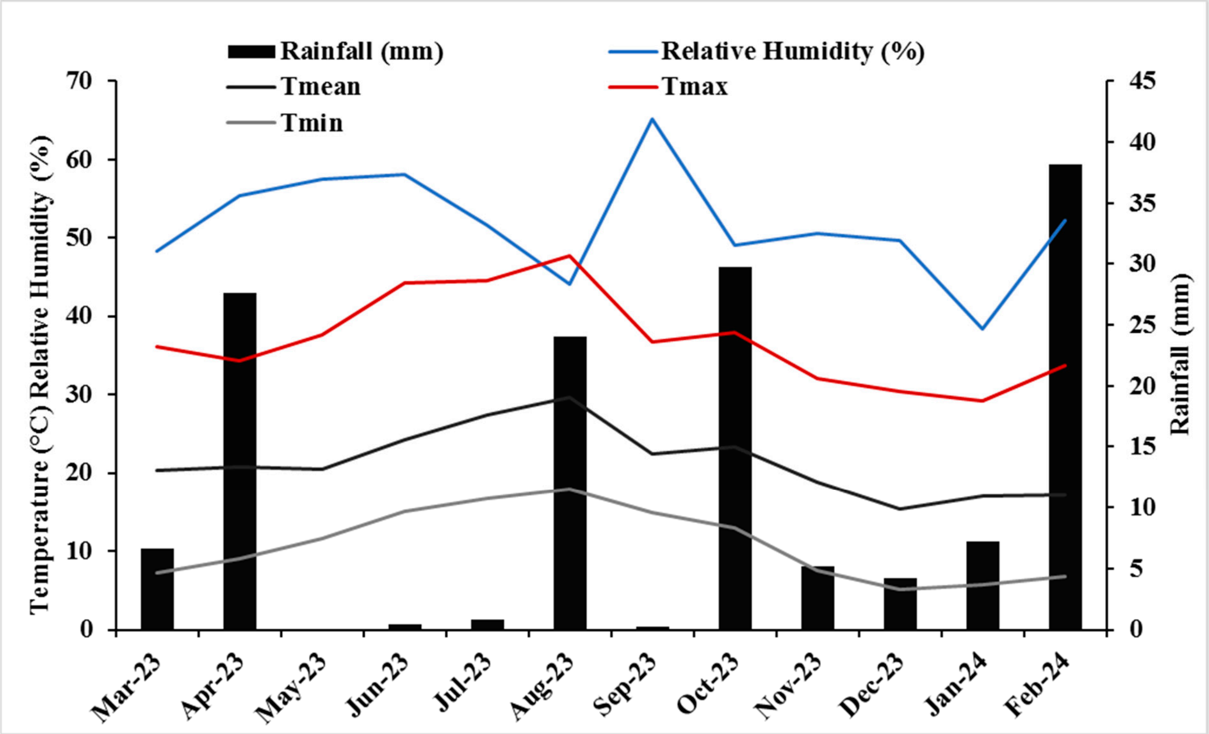


**Figure S1.** Variation of the monthly precipitations, temperatures (Tmean, T max and Tmin) and relative humidity during the experiment period
